# Pathological Society of Dublin

**Published:** 1846-06

**Authors:** 


PATHOLOGICAL SOCIETY OF DUBLIN.

*Ulceration of the Small Intestines.*—Dr. Corrigan made the following communication to the Society. A patient was admitted into the Hardwicke Hospital, December 22d, 1842, with slight fever, which passed off in a few days, the principal symptoms having been referrible to a congestion of the vessels of the brain or its membranes. The patient was in the convalescent ward about fourteen days before his death, when symptoms of disease of the intestines began to appear; at first the pulse rose, and was rather hard, but soon after there was great debility and sudden emaciation; the tongue was deeply fissured, red, and dry; the skin hot, and without any sign of perspiration; the abdomen slightly tympanitic, and free from pain on pressure: there was no diarrhœa, but occasional vomiting. Emaciation progressed very rapidly, and the patient died exhausted.

On *post mortem* examination the lower part of the ileum was found extensively ulcerated, the ulcers presenting different appearances in different places, some of them being very similar to the pustules of variola; others having the appearance of a white cicatrix, as if traced out by a snail traversing the mucous surface; while others again had advanced to a complete loss of surface, although, on account of the enlargement of the mucous follicles, they formed a projection above the surrounding mucous membrane. The disease was almost entirely confined to the small intestine, a circumstance which appears to me to account for the absence of diarrhœa, this being a symptom which I have observed only in those cases where the colon and cæcum are engaged in the disease.

These cases, coupled with a case of maculated fever in which death had occurred in six days, and which on examination presented no lesion whatever, possess much interest; and from a candid review of them it is difficult to avoid coming to the conclusion that disease of the intestinal tube does not constitute the pathology of fever.

*Cancer of the Stomach, with Adhesion to the Diaphragm and Liver.* Mr. O'Ferrall made the following communication:—A female whose countenance during life was very expressive of the existence of malignant disease, was for some time under my care, not having any obvious symptoms at all proportionate to the extent of the lesions discovered in the examination after death, which were as follows:—A large soft cancerous tumour exhibiting the cauliflower aspect, with a central depression, occupied the lesser curvature of the stomach, encroaching slightly on the cardiac orifice, but leaving the pyloric intact. Externally the stomach was adherent to the left lobe of the liver, which was there beginning to be disorganized, and it was also connected to the diaphragm, to which the convex surface of the liver was adhering; the greater part of the stomach lay under and was connected to the left lobe of the liver. The symptoms during life were principally referrible to the bowels; there was obstinate diarrhœa of six months' duration, but there was neither vomiting nor pain in the stomach. The only indication of the stomach being affected was, that after taking food there was a sensation described as resembling the sudden stoppage of the morsel immediately after its being swallowed. There were two species of such cases liable to escape detection; in one there was stricture of the pylorus; the other was without that complication; these were distinguishable from each other by very attentive observation. The present case might have been mistaken for an abdominal tumour, but the adhesion to fixed parts, such as the liver and the diaphragm, prevented the formation of any tumour; it would have been otherwise if the cancer were situated in the greater curvature. A case of this kind has occurred in which an adhesion had been formed to the colon, and an opening led from the stomach into that intestine, about which there were numerous peritoneal adhesions, and an immense deposition of cancerous matter. The contents of the stomach, for instance, and pills, retaining their silvering, then passed at once into the great intestine. In this form of the disease shown in the present specimen, there is no disease of the omentum.

*Stricture of the Rectum.*—Dr. Kirkpatrick exhibited the pellicle viscera of a female who died of acute disease while labouring under stricture of the rectum. This specimen he considered interesting, as an instance of a class of cases occasionally met with where disease situated in the rectum gives rise to symptoms of urinary irritation, causing much difficulty in diagnosis. The patient, Anne Hume, aged 45, came under his notice two years since, labouring under well marked symptoms of catarrh of the bladder. She had great and constant pain, with frequency in making water, which was alkaline, and deposited a copious sediment. In making an accurate examination of the bladder, the finger was introduced into the rectum, and accidentally discovered a close and firmly resisting stricture. The patient had made no complaint of it, nor was she aware of any disease in that situation. A simple treatment of anodyne enemata, lenitive electuary, and mucilaginous drinks, was followed by the complete removal of the urinary symptoms; the disease in the rectum giving little annoyance up to the period of her death, which took place from acute chest disease. The bladder was perfectly healthy; the orifice of the anus presented the peculiar ewer-lip appearance described by Mr. Colles; two inches above it the stricture was observed: it scarcely admitted the point of the finger. All the coats of the intestine were thickened, but the stricture itself did not occupy much extent, terminating in a sharp edge internally. Above, the intestine was not dilated, but there was superficial ulceration of the mucous membrane; below the stricture there was no ulceration. This disease had nothing of a malignant character, but was a specimen of the simple organic stricture. Dr. K. mentioned that he had lately met another remarkable case in a male patient, who had presented well-marked symptoms of urinary disease during three years; had been in several hospitals, and frequently sounded, as he had some symptoms of stone, viz., bloody urine, and pains in the glans penis. This patient died lately, and his bladder was found in a perfectly healthy condition, but an ulcer existed in the rectum, communicating with a large abscess between it and the bladder.—*Ibid.*

